# Cross-sectional analysis of circulating tumor DNA in primary colorectal cancer at surgery and during post-surgery follow-up by liquid biopsy

**DOI:** 10.1186/s13046-020-01569-z

**Published:** 2020-04-20

**Authors:** Matteo Allegretti, Giuliano Cottone, Fabio Carboni, Ettore Cotroneo, Beatrice Casini, Elena Giordani, Carla Azzurra Amoreo, Simonetta Buglioni, Maria Diodoro, Edoardo Pescarmona, Settimio Zazza, Orietta Federici, Massimo Zeuli, Laura Conti, Giovanni Cigliana, Francesco Fiorentino, Mario Valle, Patrizio Giacomini, Francesca Spinella

**Affiliations:** 1 Oncogenomics and Epigenetics, IRCSS Regina Elena National Cancer Institute, Via Elio Chianesi, 53, 00144 Rome, Italy; 2 Oncogenomics Division, Eurofins Genoma Group, Via Castel Giubileo, 11, 00138 Rome, Italy; 3 Digestive Surgery, IRCSS Regina Elena National Cancer Institute, Rome, Italy; 4 Pathology, IRCSS Regina Elena National Cancer Institute, Rome, Italy; 5 Medical Oncology 1, IRCSS Regina Elena National Cancer Institute, Rome, Italy; 6 Clinical Pathology, IRCSS Regina Elena National Cancer Institute, Rome, Italy

**Keywords:** Colorectal carcinoma, Liquid biopsy, Circulating tumor DNA, Next generation sequencing, Digital PCR

## Abstract

**Background:**

Liquid biopsy (LB) in early-stage, non-metastatic colorectal cancer (CRC) must be sensitive enough to detect extremely low circulating tumor DNA (ctDNA) levels. This challenge has been seldom and non-systematically investigated.

**Methods:**

Next generation sequencing (NGS) and digital PCR (dPCR) were combined to test tumor DNAs (tDNAs) and paired ctDNAs collected at surgery from 39 patients, 12 of whom were also monitored during the immediate post-surgery follow up. Patients treated for metastatic disease (*n* = 14) were included as controls.

**Results:**

NGS and dPCR concordantly (100% agreement) called at least one single nucleotide variant (SNV) in 34 tDNAs, estimated differences in allelic frequencies being negligible (±1.4%). However, despite dPCR testing, SNVs were only detectable in 15/34 (44.1%) ctDNAs from patients at surgery, as opposed to 14/14 (100%) metastatic patients. This was likely due to striking differences (average 10 times, up to 500) in ctDNA levels between groups. NGS revealed blood-only SNVs, suggesting spatial heterogeneity since pre-surgery disease stages, and raising the combined NGS/dPCR sensitivity to 58.8%. ctDNA levels at surgery correlated with neither tumor size, stage, grade, or nodal status, nor with variant abundance in paired tDNA. LB sensitivity reached 63.6% when ctDNA was combined with CEA. Finally, persistence and absence of ctDNA on the first conventional (month 3) post-surgery follow-up were associated with fast relapse and a disease-free status in 3 and 7 patients, respectively.

**Conclusions:**

A simple clinical NGS/dPCR/CEA combination effectively addresses the LB challenge in a fraction of non-metastatic CRC patients.

## Background

Liquid biopsy (LB) interrogates blood for trace amounts of circulating analytes such as circulating tumor DNA (ctDNA), miRNAs, exosomes and tumor cells. Taking advantage of next generation sequencing (NGS) and digital PCR (dPCR), LB is being increasingly applied to a variety of human tumors, including colorectal carcinoma (CRC) [1–7]. Peculiar of CRC, different DNA analytes find elective application at different disease stages. Methylated DNA is preferred to predict CRC likelihood in pre-symptomatic and symptomatic population [8]. ctDNA is instead preferred in metastatic CRC (mCRC), since it is abundant and easily detectable when tumor burden is high [9–13]. For instance, a clinical trial in 115 patients with mCRC demonstrated an excellent agreement in the RAS mutational status between tissue and plasma, strongly supporting the application of ctDNA to assign EGFR-blockade therapy [13]. However, the catalogue of actionable mutations is steadily expanding, and target therapies are being applied in progressively earlier (e.g. adjuvant) settings [14]. Thus, ctDNA may readily become an appealing alternative/adjunct for early CRC detection and management in a wide applicative niche spanning from pre-symptomatic to advanced cancer.

Whereas a large number of studies have addressed mCRC [15–23] only a few have investigated the role of liquid biopsy in early, non-metastatic CRC patients at surgery, e.g. in conditions of extremely low tumor burden [24–28]. Reinert et al. [26] applied genome-wide sequencing to a small pilot cohort. Phallen et al. [27] implemented a targeted error correction sequencing (TEC-Seq) approach on 27 CRC patients at stages I/IV, surprisingly reporting very similar variant alleleic frequencies (VAFs) and assay sensitivities across CRC groups. Finally, Cohen et al. [28] developed CancerSEEK, a PCR-based assay for frequently mutated hotspots integrated with protein biomarkers, scoring positive in about 65% of patients at surgery. Of interest, none of the cited studies was designed to provide in the same place quantitative ctDNA comparisons between patients bearing minimal, accurately estimated tumor burdens on the one hand, and patients bearing metastatic CRC on the other. In addition, it remains unclear whether any tumor size threshold applies, e.g. whether mutations in the blood can be detected only above certain primary tumor sizes. Moreover, one may wonder whether LB might complement information obtained with conventional clinical pathological parameters of the primary tumor and/or circulating biomarkers such as the carcinoembryonic antigen (CEA) and Ca19.9. Finally, accurate comparisons of two major, widely available LB techniques such as NGS and dPCR are limited to mCRC patients [29]. Addressing all these issues in a single case collection may provide insight into ctDNA release from small primary tumors, and may ultimately be useful to improve/accelerate clinical decisions in the immediate post-surgery follow-up period and in real-life settings.

On these premises, we decided to gather quantitative information on ctDNA at surgery and immediately thereafter focusing our attention on non-metastatic patients with primary CRC lesions not exceeding 7 cm in their major diameter. All patients were free of previous treatments, and were enrolled at surgery, days to weeks after being informed of their clinical diagnosis. This specific time of recruitment is optimal to ask all the above questions, since total-body clinical imaging excludes in most patients the presence of metastatic foci. In these patients, tumor burden may be most accurately defined upon clinical-pathological examination of surgical specimens. Thus, paired tumor and blood samples provide an accurate tDNA/ctDNA cross-sectional snapshot at a time point when tumor burden is known with considerable accuracy, although cryptic micrometastatic foci cannot be obviously excluded. In the context of a technical validation of state-of-art LB methods foreseen by a multinational H2020 EU project (www.ultraplacad.eu) tDNAs and ctDNAs were simultaneously interrogated by clinical NGS and dPCR. In a subgroup of patients, ctDNA levels were also monitored during the immediate post-surgery follow-up period and during chemotherapy. As described herein, multiplatform cross-sectional testing and follow-up suggest ways to improve perioperative LB and its application to non-metastatic, newly diagnosed CRC, taking advantage of simple tools that are available to most clinical sequencing facilities.

## Materials & methods

### Patients and biological samples

Three sets of patients were enrolled in the present study after signing a written informed consent. Group A comprises prospectively collected (November 2015 to January 2019) consecutive patients (*n* = 39) admitted to surgery for removal of their primary CRC after exclusion of extra-colonic metastatic involvement by medical imaging. Blood and tissue samples were obtained on the same day, before and during surgery, respectively. Group B patients are the first 12 consecutive patients from group A who were enrolled for serial blood drawing on the occasion of post-surgery follow-up. Group C comprises distinct patients (*n* = 14) bearing metastatic CRC. Primary CRC tissues of these patients were obtained from the institutional BioBank. All samples were anonymized, and the laboratory personnel was double-blinded with regard to clinical pathological data. Demographics and clinical pathological features are presented in Tables S1 and S2.

### DNA extraction

Sections (5 μm-thick) were cut from a representative formalin-fixed, paraffin-embedded (FFPE) tissue block. One section was counterstained by hematoxylin/eosin and assessed for quality (tumor fraction ≥50%) by an expert pathologist. Sections were then deparaffinized and digested overnight at 56 °C with proteinase K (Qiagen, Hilden, Germany). DNA was extracted by the QIAmp DNA FFPE Tissue Kit (Qiagen) according to the manufacturer’s instructions, and aliquoted in three different vials, each of which was given to three different users for testing in the two NGS platforms and dPCR. Blood (20 ml) was drawn in BD Vacutainer K_2_EDTA tubes and processed within 1 h. Plasma was isolated by two successive rounds of centrifugation at 4 °C (2000 x *g* for 20 min, and 16,000 x *g* for 10 min), and stored at − 80° in single-use 2 ml aliquots until circulating free DNA (cfDNA) extraction. No freeze-thawing cycles were allowed. cfDNA was extracted by the QIAmp circulating nucleic acid kit (Qiagen) according to the manufacturer’s instructions in a final volume of 30 μL, and stored at − 20 °C until analysis. Both tDNAs and cfDNA were fluorimetrically quantified with the Qubit dsDNA HS assay kit (Life Technologies, Carlsbad, CA, USA).

### Library preparation and sequencing

NGS was performed in parallel at IRE, a non-profit public health provider, and at LabGen, a for-profit private molecular diagnostic laboratory. At IRE, libraries from 10 ng of tDNA were prepared with the Ion AmpliSeq™ Library kit 2.0 and the Colon and Lung Panel (Life Technologies), as per manufacturer’s instructions. The panel encompasses 504 hotspot regions in 22 genes. Libraries were then automatically loaded onto the Ion 530 chips by the Ion Chef system (Life Technologies). After sequencing on Ion S5, data were analyzed with the Ion Reporter suite version 5.6 (Life Technologies). A custom filter chain including restriction on location (exonic), *p* value (< 0.05), variant effect (unknown, missense, nonsense, stoploss, frameshift insertions and deletions), variant type (SNV, small INDELS, MNV), filtered coverage (*n* = 200) and VAF ≥4% was applied to filtering NGS data. At LabGen, 10–40 ng of tDNA and ctDNA were used to prepare libraries with the TruSight Tumor 15 panel (Illumina, San Diego, CA, USA), according to the manufacturer’s protocol. Quality assessment and quantification of libraries were performed with the D1000 Screen Tape system (Agilent, Santa Clara, CA, USA) and the Qubit dsDNA HS assay kit (Life Technologies), respectively. Libraries were sequenced on the MiSeq® (tDNA) and on the NextSeq® (ctDNA) sequencing systems (Illumina) in a paired-end configuration (2 × 150 bp). Data were analyzed using the on-board Reporter 2.6.2 software. For tDNA analysis, a minimum of 500x coverage was required on > 93.5% of the bases targeted by the assay for variant calling occurring at frequencies as low as 4%. For ctDNA analysis, to pass quality control for variant, a minimum of 25.000x coverage was required on > 99.8% of amplicons. NGS data were filtered using a custom data interpolation pipeline for in silico validation, resulting in a confidence VAF threshold value of ≥0.2%. Selected tissue samples were also re-sequenced by applying the same pipeline of ctDNA to identify whether mutations detected only in the bloodstream were present in the corresponding tDNA at frequencies lower than 4%. All NGS variants were manually reviewed with Integrative Genomics Viewer (IGV version 2.2, Broad Institute, Cambridge, MA, USA) and Biomedical Genomics Workbench Version 4.0 (Qiagen), and then matched against the ClinVar (https://www.ncbi.nlm.nih.gov/clinvar/) and COSMIC (https://cancer.sanger.ac.uk/cosmic) databases. Results were separately elaborated at IRE and LabGen, and collected by a third investigator for dPCR assay design.

### Digital PCR

Primers and probes were designed with the Custom Taqman® Assay Design Tool (CADT, Life Technologies). Matched tissue and blood samples from each patient and cfDNA from healthy donors were run in the same experiment using the chip-based QuantStudio™ 3D Digital PCR System (Life Technologies). Reactions were set up in a final volume of 16 μl including 8 μl of 2x Master Mix, 0.9 nM of each forward and reverse primers, 0.25 nM of TaqMan® MGB probe, 7.0 μl of template, and loaded onto dPCR chips. Input DNA for tissue analysis was normalized to 20 ng. By contrast, input cfDNA was equalized by plasma volume (0.5 mL) to accurately measure ctDNA copies/mL. Thermal cycling was as follows: 10 min at 96.0 °C, 39 cycles at 56.0 °C for 2 min, 30 s at 98.0 °C, and a final elongation step of 2 min at 60 °C. Threshold values of FAM and VIC fluorescence were automatically calculated by the Thermo Fisher Cloud Analysis Suite in tissue samples, manually reviewed, and then applied to the corresponding ctDNA. The complete list of commercial and custom-designed dPCR assays is presented in Table S3.

### Circulating biomarkers

Blood samples (5 ml) collected from the cubital vein were assessed for serum CEA and Ca19.9 levels by an electrochemiluminescence immunoassay on the Cobas e801 immunoassay analyzer (Roche Diagnostics, Mannheim, Germany). The specimen was automatically diluted if exceeding the upper limit value. As per manufacturer’s instructions, cut-off values (positive vs negative) were set at 4.7 ng/ml and 34 U/ml for CEA and Ca19.9, respectively.

### Specificity controls and statistical methods

Three different negative controls were included to assess assay specificity: (a) healthy donors (*n* = 10) from the Institutional blood transfusion centre; (b) plasma samples pooled from 5 healthy donors (5 different batches); (c) in the case of dPCR, plasma samples from group A patients were scrambled and re-assorted to use ctDNAs to prime dPCR assays for SNVs absent in that specimen. None of these negative controls met the criteria for variant calling. Sensitivity and specificity of LB were calculated as described [30] (Table S4). VAF values in tumor tissue and blood were correlated by regression analysis. Descriptive statistics were used to summarize patients and disease-relevant features. The Fisher’s exact test was used to calculate associations between clinical pathological parameters and ctDNA abundance. Two-sided *p* values < 0.05 were considered statistically significant. SPSS software (v.22, SPSS Inc., IL, USA) was used for statistical elaborations.

## Results

### Study design

**Fig. 1:**
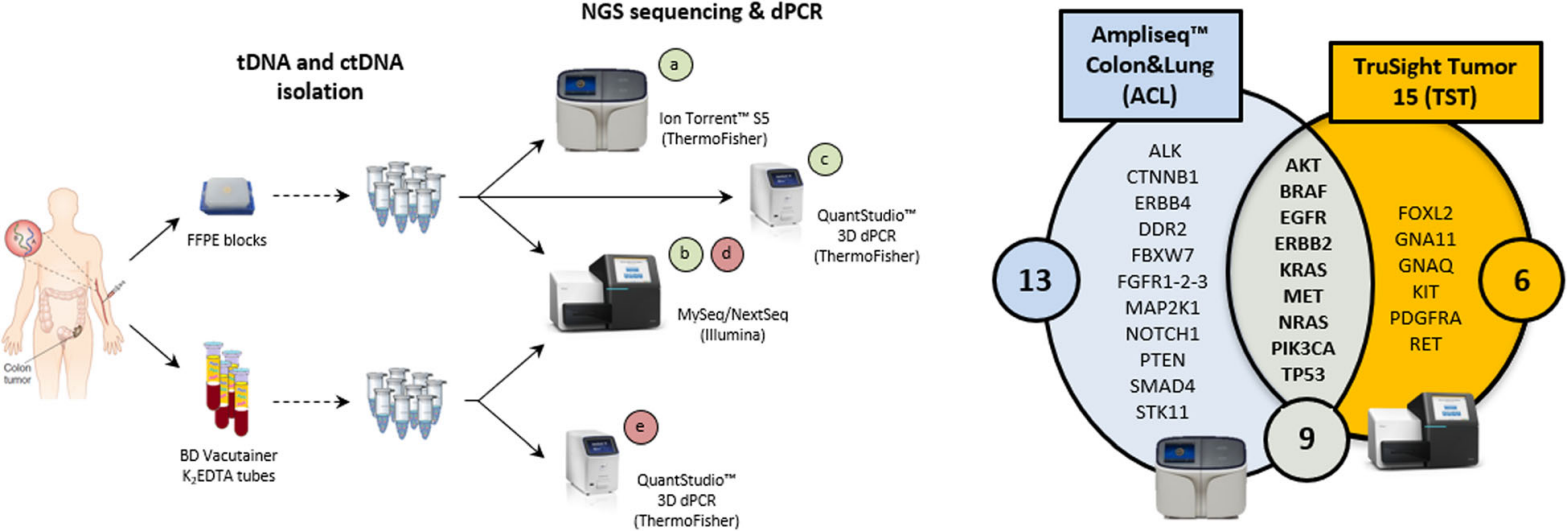
Study flowchart. Tissue and blood samples from CRC patients bearing primary tumors were collected on the day of surgery, and processed to obtain tDNAs and ctDNA, respectively. Lab staff was double-blinded, and all tDNAs were analyzed by two different NGS platforms, e.g. the ThermoFisher Ion Torrent S5 with the ACS targeted *NGS* panel (a), and the Illumina MySeq with the TST targeted *NGS* panel (b). Gene panels and their overlap are shown (right). Selected mutations were then validated by the QuantStudio 3D dPCR system (c). Sequencing was by NextSeq also in the case of ctDNAs (d); validation of selected mutations was by dPCR (e)

This study was primarily designed to analyze paired tissue and blood samples obtained within hours from each other on the day of surgery. The same paired scheme was applied to post-surgery follow-up and metastatic CRC patients. For stringent testing, paired tDNA/ctDNA samples were assessed by both targeted NGS and dPCR. The former was used to interrogate for defined sets of cancer alterations. The latter was used to confirm SNVs, and for ultra-sensitive ctDNA detection. As shown in Fig. 1, tDNAs were tested by two targeted NGS panels (Ampliseq™, ACL, and TruSight™, TST) and by dPCR (a-c), whereas ctDNAs were tested by one NGS panel (TST) and dPCR (d and e). Since 9/28 tested genes are common to the two NGS panels (Fig. 1, right), and dPCR assays are custom-designed on the basis of NGS, there is a large double to triple overlap area of data collection and technical comparison among ACL, TST and dPCR calls.

### Identification of SNVs in tumor tissues at surgery

NGS data filtering of tDNAs from group A patients returned a list of 54 hits (44 and 48 detected by TST and ACL, respectively) comprising 39 distinct somatic SNVs. No mutations were detected in 5/39 patients (12.8%). SNVs in the 34 positive patients were equally distributed between single (*n* = 17) and multiple (*n* = 17) hits (Fig. S1). A specific dPCR assay was designed to test at least one SNV (detected by either NGS platform) in each of the 34 tDNAs. Remarkably, not only variant calls but also mean VAFs were concordantly assigned in the double or triple ACS/TST/dPCR overlap areas, e.g. 33.0% (SD ±1.3%) for NGS (the 2 platforms altogether), and 33.1% (SD ±1.4%) for dPCR. In summary, accurate study design resulted in assay-independent variant calling and VAF estimates, which prompted us to apply these techniques to ctDNA.

### Quantitative differences in ctDNA levels at surgery and in metastatic patients

**Fig. 2:**
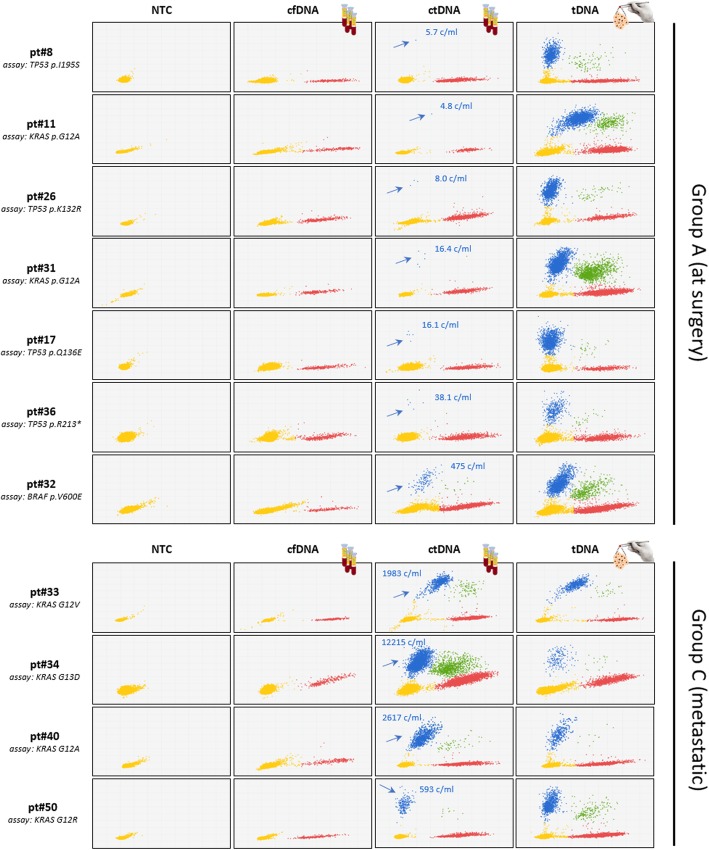
dPCR analysis of ctDNAs at surgery and from patients bearing metastatic CRC. ctDNAs/tDNA pairs from representative patients (at surgery and metastatic, upper and lower panels, respectively) were assessed by dPCR. Different input DNAs were used in different rows: NTC, no template control; cfDNA, circulating free DNA from healthy donors; ctDNAs and tDNAs, paired tumor and plasma samples from any given patient. Group A patients are arranged top to bottom in order of increasing pathological staging and grading. Yellow, red, blue and green dots depict not amplified, WT, MUT and double-positive dPCR spots, respectively. Red, blue and green dots altogether: cfDNA. Blue and green dots: ctDNA. ctDNA copies per mL are noted

To accurately estimate differences in ctDNA levels between patients with undetectable (at surgery) and clinically proven metastatic spread, the 39 SNVs plus 2 additional SNVs similarly detected by NGS in the primary tumors of group C patients were tested by dPCR. Two templates were used in parallel: (a) ctDNA from the equivalent of 0.5 mL of plasma, and (b) 20 ng of genomic DNA from the paired tumor tissue (tDNA). tDNA served as an internal reference for SNV tissue abundance. Representative dPCR plots (Fig. 2) show that ctDNA is far less abundant at surgery than in metastatic patients, as seen by comparing upper (arrows) and lower panels. Mean ctDNA copy numbers were: 57.8 ± 123.5 copies/mL, ranges 2.8–475.0 in group A vs 2313.9 ± 4119.7 copies/mL, ranges 21.8–12,215.3 in group C. Mean VAF values were 1.4 ± 1.8%, ranges 0.1–7.0% in group A vs 21.2% ± 18.5%, range 2.8–55.0% in group C, resulting in a > 1 log difference in ctDNA copy number between the two groups. The striking ctDNA enrichment associated with metastasis is readily appreciated if one considers that in some of these patients (e.g. pt.#34 and pt.#40) 0.5 mL of plasma contain far more mutated DNA than 20 ng of concentrated DNA from the corresponding primary tumor lesion, the latter estimated to correspond to about 3000 copies of diploid human genomes. Also, the lowest and highest outliers in groups A and B differed > 500 times. Altogether, these observations provide evidence for dramatic ctDNA load in conditions of high tumor burden and exemplify the considerable technical challenge of performing LB at surgery.

### Detection of ctDNA at surgery: a combined NGS/dPCR approach

Matching tDNA/ctDNA sequencing data pairs (at or above their respective pre-set LODs) identified a subset of SNVs that can be detected in tDNA/ctDNA pairs by NGS, dPCR, or both. Data from this subset of 20 ctDNA^+^ are analytically displayed in Fig. S2, and the entire 34 patient dataset is summarized in Table 1. SNVs shared between tissue and blood are referred to as ‘type (a)’ SNVs. Altogether, NGS and dPCR could detect 12/44 (27.3%) and 17/34 (50.0%) type (a) SNVs in 9/34 (26.5%) and 14/34 (41.2%) patients with at least one testable tissue SNV, respectively. The greater fraction of tissue SNVs detected by dPCR was entirely accounted for by a set of 6 SNVs in 5 ctDNAs that could be seen by dPCR but not NGS (Table 1, left side). Remarkably, when this testing scheme was applied to group C patients, all of them were scored LB-positive. At the same time, it became readily evident that NGS of blood taken on the day of surgery was able to detect 12 circulating SNVs distinct from those seen in tissues (Fig. S2). Taking these ‘type (b)’ SNVs into account brings the total number of NGS-positive ctDNAs to 15/34 (44.1%; Table 1, right side). This observation was intriguing, since the primary tumor is expected to be the main or only source of ctDNA in patients who have no medical imaging evidence of extra-colonic tumor dissemination. Indeed, paired tDNA re-sequencing with data filtering at VAFs < 4%, and dPCR testing confirmed that at least some (3/12) of these SNVs were present in tissues (Table 1 and not shown). Therefore, type (b) SNVs may originate from minor tumor subsets (spatial heterogeneity) within the sampled area of the primary tumor, and possibly also from un-sampled, ‘cryptic’ tumor sites [31]. Nevertheless, when type (a) and type (b) SNVs altogether are cumulatively considered, the number of LB-positives increases to yield an overall assay sensitivity of 20/34 (58.8%) of CRC patients at surgery (Table 1, lower right box). These results demonstrate that even small commercial NGS panels capture a sufficient number of SNVs to be applicable to most CRC patients in the bona fide non-metastatic, low-ctDNA setting of CRC patients at surgery. Combining NGS and dPCR enhances LB sensitivity.

**Table 1:** Summary of somatic alterations (SNVs) detected in tDNA and ctDNA

SNVs	(a) shared between tissue and blood		(b) blood only	(a) + (b)
	type (a) SNVs	patients with type (a) circulating SNVs	type (b) SNVs	patients with types (a) and (b) circulating SNVs
NGS (TST)	12/44 (27.2%)	9/34 (26.5%)	12	15/34 (44.1%)
dPCR	17/34 (50.0%)	14/34 (41.2%)	1	15/34 (44.1%)
NGS + dPCR	17/34 (50.0%)	14/34 (41.2%)	12	20/34 (58.8%)

### Performance of LB assays

The performance of LB assay (NGS and dPCR combined) at surgery was assessed on group A CRC patients and 10 healthy donors (Table S4). Both the positive predictive value and specificity of the assay were 100%, since there were no detectable circulating mutations were found in healthy donors, whereas the negative predictive value was 34.5%, which reflects the 19 CRC cases in which no circulating SNV was detected. Sensitivity, e.g. the fraction of true positives, was 51.3% and accuracy was 61.2%. These results demonstrate that a mutational status, when detectable in blood, correlates tightly with a CRC-bearing status, although negative data remain uninformative.

### ctDNA levels at surgery poorly correlate with tDNA abundance and clinical pathological features

**Fig. 3:**
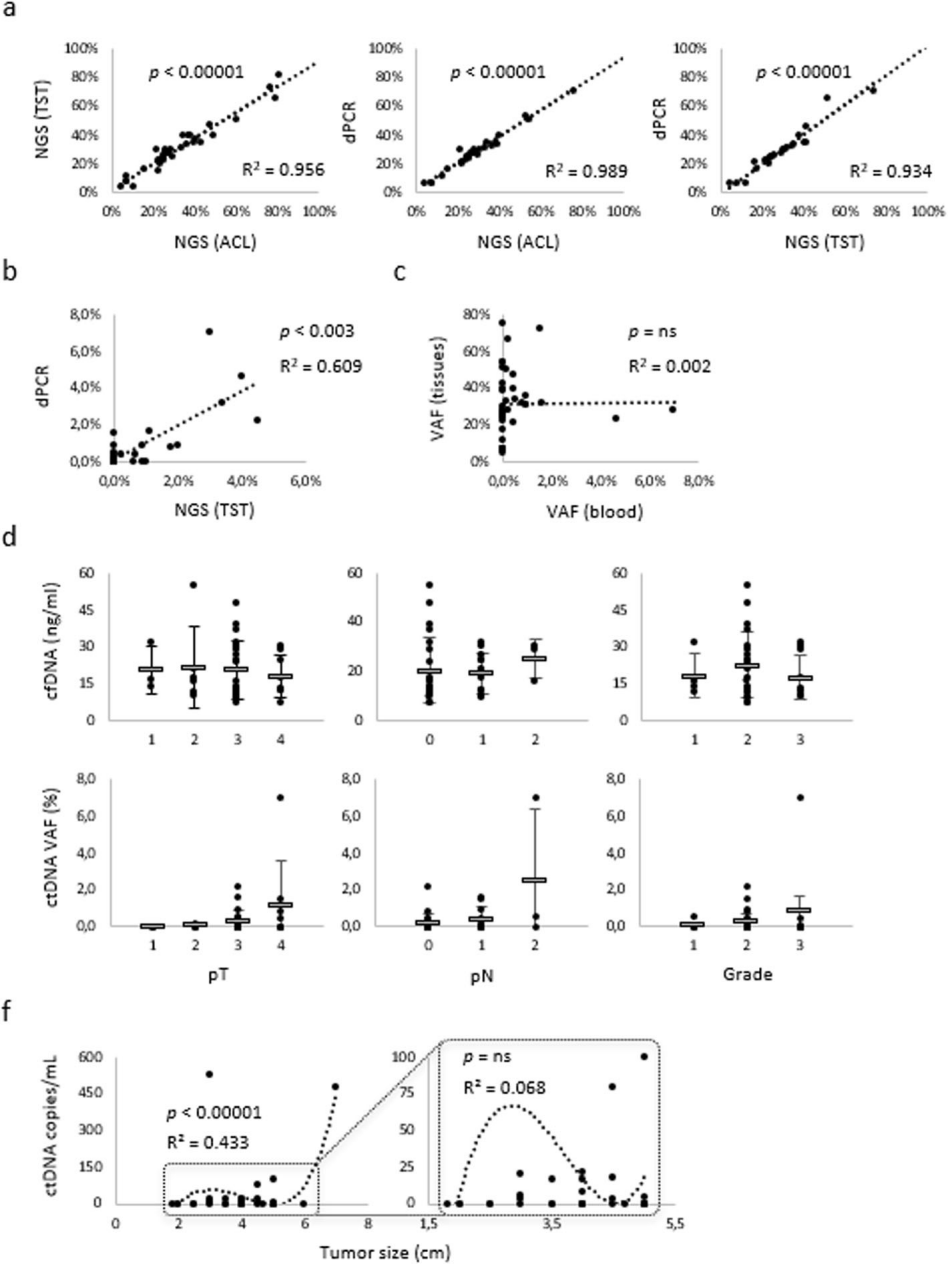
Correlation statistics. **a** Tissue DNAs were tested by NGS (ACS and TST panels) and dPCR. VAF values were calculated within the 9-gene NGS panel overlap, and then paired two by two in the three regression plots, as indicated. Additional plots were generated by pairing VAF values from (**b**) ctDNAs (NGS vs dPCR), and (**c**) tDNA/ctDNA (dPCR vs dPCR) from individual patients. ctDNA levels assessed by dPCR were correlated with (**d**) pathological tumor staging and (**e**) tumor size (major diameter). In (**e**), the best fit is defined by a polynomial curve, and the area in the low copy number range is expanded to enhance resolution of low tumor sizes. Regression coefficients and significance (Student’s T-test) are shown. Abbreviations: ns, non-significant

Next, we sought to assess correlations, if any, between ctDNA levels in group A patients on the one hand, and tDNA abundance or clinical pathological variables of their paired primary tumors, on the other. Preliminarily, we noted that linear regression analysis of VAFs obtained by the three different testing methods (ACS, TST and dPCR) resulted in striking regression coefficients in the case of tDNAs (Fig. 3a), providing evidence for inter-assay consistency. However, when the analysis was repeated on ctDNAs, correlations were far less evident, although still significant (Fig. 3b). Regression drop-off was not due to trivial technical reasons, but to the 6 dPCR^+^/NGS^−^ paired values (noted in Table 1, first column) that push the regression line toward the ordinates. Then, we resorted to dPCR for further elaborations. Firstly, we asked whether SNV abundance in tissue might correlate with SNV representation in blood. No linear relationship was apparent by tDNA/ctDNA pairing of dPCR VAF values (Fig. 3c), demonstrating that factors other than VAF in tumor tissues may regulate release and accumulation of ctDNA in blood. Secondly, we asked whether total cfDNA and ctDNA correlate with pT, pN and pathological grade. This was certainly not the case for cfDNA (Fig. 3d), whereas weak, non-significant trends could be exclusively seen between ctDNA and pT (Fig. 3e). These results suggest weak ctDNA/staging correlations at best. Lastly, ctDNA levels were correlated with tumor size (ranges from 3 to 7 cm in diameter along the major axis). Again, there was no significant linear regression, but we were able to fit the data by a polynomial, biphasic curve with a distinct inflection at the 4.7 cm size (Fig. 3f, left). Above this threshold ctDNA and tumor size were roughly proportional, but when the part of the graph below inflection was expanded (Fig. 3f, right), the dots representing this set of smallest tumors became scattered in an essentially random fashion. This is consistent with ctDNA levels becoming unrelated to tumor size particularly in the low dimension bracket, 4.7 cm roughly marking the ctDNA/size non-correlation threshold. Along this line, no ctDNA was detectable in the 3 patients with tumors < 3 cm.

### Correlations between ctDNA and circulating CEA or Ca19.9

**Fig. 4:**
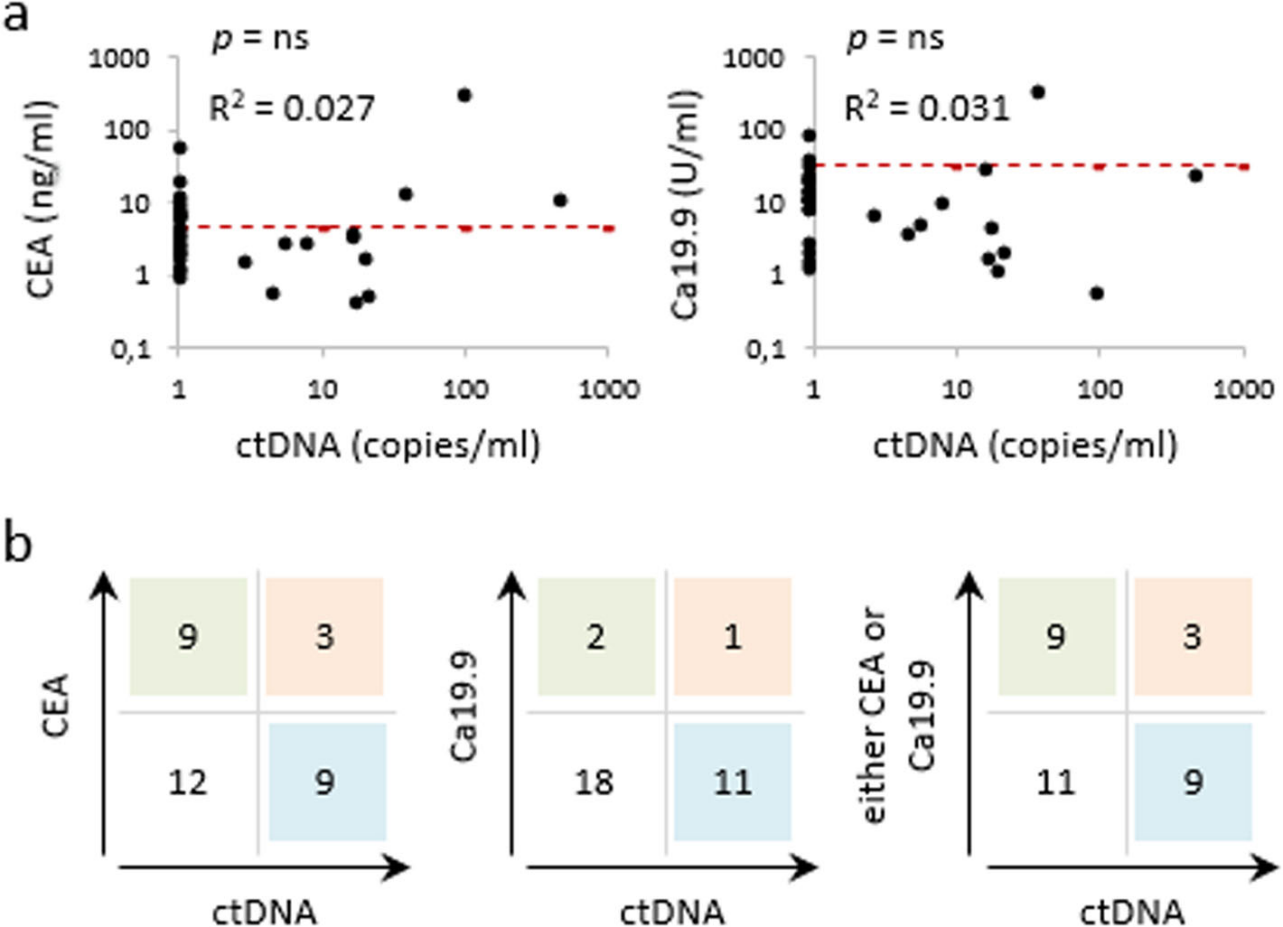
Comparison between conventional serum biomarkers and circulating DNA. **a** Regression analysis of ctDNA dPCR values on the day of surgery paired with serum CEA and Ca19.9 levels assessed in the 15 days prior to surgery. All values are in logarithmic scale. Regression coefficients and *p* values are shown. ns: nonsignificant. Red dotted lines: CEA and Ca19.9 positivity threshold (4.7 ng/ml and 34 U/ml, respectively). Samples with undetectable ctDNAs are aligned to the Y axis. **b** A scoring matrix was built to match dPCR positives (any value) and negatives (undetectable) on the one hand vs CEA and Ca19.9 positives and negatives on the other

Since serum biomarkers are a mainstay of CRC management, we sought to compare and integrate plasma ctDNA with CEA and Ca19.9. To this end, we focused on a subset of patients who had undergone a CEA and/or Ca19.9 blood test in the 15 days preceding surgery/ctDNA testing. In this group A subset we were unable to find significant correlations between ctDNA and serum biomarker levels (Fig. 4a). In addition, Ca19.9 was poorly informative, since it rarely exceeded the positive threshold value. When single and double positives of ctDNA, CEA and Ca19.9 were enumerated in a scoring matrix (Fig. 4b), it became readily apparent that CEA and ctDNA identify largely non-overlapping patient populations of the same size (*n* = 9 in both cases) with only 3 double positives. As a result, single assessment by either CEA or ctDNA correlated with a tumor-bearing status in 12/33 (36.4%) patients, whereas the two altogether identified 21/33 (63.6%) patients. Thus, factoring ctDNA and CEA into a single score may provide incremental information as to disease status.

### ctDNA follow-up in the post-surgery and advanced disease settings

**Fig. 5:**
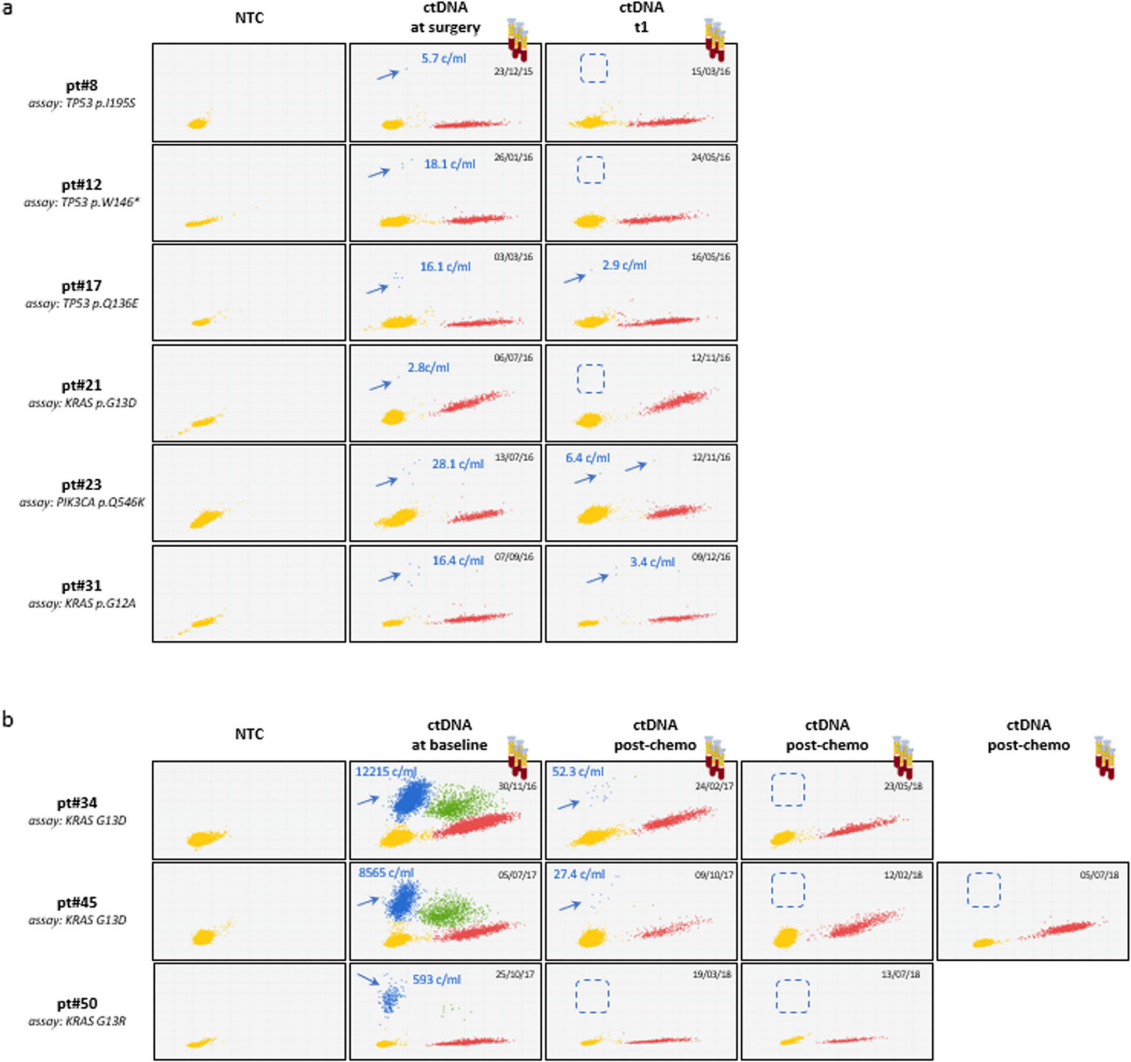
Liquid biopsy during clinical follow-up. **a** Representative results of ctDNA levels at surgery and 3 months after surgery (t_1_). **b** Three examples of ctDNA responses to chemotherapy, apparently complete. Progressive disappearance of ctDNA may be appreciated in boxed areas. Abbreviations: NTC, no template control

As per international guidelines, routine post-surgery CRC follow-up is carried out by clinical examination, laboratory tests and CT scans at month 3 after surgery (t_1_), and then at 3-month intervals. A subset of group A patients (group B) was scheduled for dPCR ctDNA assessment at t_1_. All 3 patients with ctDNA present at surgery and persisting at t_1_ recurred very rapidly, as documented since the very first routine CT scan. In contrast, no relapse/progression was seen by clinical, radiological and serological criteria in the 7 patients with no detectable ctDNA at t_1_, regardless of whether the ctDNA had (*n* = 4) or had not (*n* = 3) been detected at surgery. The observation period was variable, ranging from 6 to 40 months (median: 11.7 months). Two patients were lost to follow-up. LB was also useful to monitor response to therapy in metastatic patients (group C), as shown by dramatic ctDNA drops in association with chemotherapy (Fig. 5b). In these patients LB takes advantage of much higher baseline ctDNA levels and operates over a much higher dynamic range of ctDNA loads, highlighting once again the challenge of LB in the immediate post-surgery setting.

In summary, although the small numbers do not allow drawing firm conclusions, these results altogether provide evidence that ctDNA is associated with CRC response to surgery and systemic therapies in the immediate post-surgery and metastatic settings, respectively.

## Discussion

In the present report, we have carried out a stringent, comparative technical evaluation of the merits and limitations of NGS and dPCR, using two distinct NGS platforms and a series of dPCR assays detecting selected SNVs. Remarkably, NGS and dPCR concordantly called (100% agreement) SNVs in tumor tissues, and displayed an inter-assay VAF variability only slightly above 1%. We approached LB with these validated tools, and asked a series of questions that had not been systematically addressed before and are discussed one by one in the paragraphs below.

### A comparison of ctDNA at surgery and after metastatic spread

As compared to patients with metastatic CRC, patients at surgery displayed ctDNA concentrations 10-fold lesser on average. When the extreme ctDNA outliers are considered, the gap between the lowest (few copies/mL) and the highest ctDNA (hundreds of copies/mL) concentrations expands to an impressive 500-fold or more. These results emphasize the challenge of LB at surgery. However, ultra-low ctDNA concentrations may not be the only hurdle. The complex relationships between ctDNA on the one hand and the clinical pathological features of the primary tumor sampled on the same day hint at alternative/additional factors limiting ctDNA release/accumulation.

### Clinical pathological variables and ctDNA sources

First of all, ctDNA levels do not reflect, at surgery, the mutation frequencies (VAFs) observed in paired tissue. This implies that SNVs abundant in the tumor (including the mutations selected for our own dPCR analysis) may not necessarily candidate as optimal LB analytes. Indeed, a recent pre-clinical study by our group in mice bearing small (0.6–1.0 cm in diameter) CRC tumor xenografts [32] showed that the abundance of ctDNA is not purely dependent on tumor size, but on the fine, mutation-specific and xenograft-dependent tuning of DNA release into the bloodstream.

The second source of poor correlation between blood and tissue is the presence of type (b) circulating alterations not readily seen in the primary tumor. Through highly sensitive tDNA resequencing we provide evidence that at least some of these SNVs originate from very small tumor cell populations within the primary tumor specimen, whereas others presumably originate from tumor regions that either were not sampled or remained ‘cryptic’ after our analysis. It may be concluded that the topography of tumor regions contributing ctDNA is complex even in bona fide single-site, non-metastatic CRC. Alternatively, some patients may already have developed systemic disease.

Thirdly, ctDNA levels at surgery display poor (if any) correlations with clinical pathological variables, including primary tumor stage and grade, as well as node status. These results are at least in part surprising because in advanced CRC ctDNA appears to be roughly related to tumor size [9–12]. However, the poor correlation detected herein is unlikely to be due to trivial technical artifacts arising from incorrect sample handling, since we adopted the same approach in a previous study on Ewing sarcoma [33] where we documented a canonical, linear correlation between circulating fusion transcripts and metabolic activity or tumor size. Herein, we found a complex, non-linear, polynomial ctDNA-tumor size relationship, that became particularly weak below a tumor size of 4.7 cm in diameter. This suggests that CRC tumors become extremely unpredictable in their ability to release ctDNA when size drops below this critical threshold. Unfortunately, published reports in early CRC provide little or no information about the relationships between ctDNA and the clinical pathological features of primary tumors, show no dimension plots, and did not look for correlations with tumor burden in most cases, making it difficult to draw detailed comparisons with the present findings. Tumor size information is instead provided by a study focused on lung cancer patients [7] in which clonal alterations (i.e. common to many cancer cells), were used for correlation analysis with tumor burden. Abbosh et al. calculated that the smallest detectable tumors would result in a mean clonal VAF of 0.1%. These LOD values are close to or slightly better than, our own estimates in CRC.

### Sensitivity and specificity of LB at surgery

In the present study, NGS captured at least one mutation in the primary tumors from 87.5% of the patients, and most SNVs detected in blood by NGS could be confirmed by independent dPCR testing. dPCR was more sensitive, in that it detected ctDNAs that would have been missed by NGS alone, but NGS was more inclusive, in that it identified type (b) mutations apparently present in blood only. As a result, either method alone disclosed a positive LB score in 15/34 (44.1%) patients, whereas their combination detected at least one SNV in up to 58.8% of testable patients at surgery, strongly indicating that NGS and dPCR should be combined for optimal performance in a clinical setting. When the entire population is considered (including patients with no detectable tissue SNVs) the combined NGS/dPCR assay detected a CRC-bearing status in the blood of the majority (21/39) of patients, essentially in the absence of false positives, yielding sensitivity and accuracy of 51.3 and 61.2%, respectively. This result was obtained taking advantage of small NGS targeted panels encompassing 25 genes altogether. In addition, although based on molecular barcoding, the TST panel used for LB was not specifically designed for low-noise variant calling. With these caveats and limitations, the reported ctDNA sensitivities by ourselves, Ciarloni et al. and Cohen et al. [25, 28] are not drastically different (58.8% vs 78 and 65%, approximately), and invariably lower than those (93%) reported in metastatic cancer [13]. We conclude that even simple LB assays, like the one described herein, may find immediate clinical application in conditions of low CRC burden.

### ctDNA and serum biomarkers

In the present study, dPCR alone and dPCR combined with CEA detected a tumor-bearing status in 36.4 and 63.6% respectively of a subset of group A patients. The considerable improvement obtained by combining ctDNA and CEA was the result of a limited overlap between ctDNA^+^ and CEA^+^ patients at surgery. Thus, ctDNA poorly correlates with yet another classical CRC readout, opening the possibility to integrate ctDNA and CEA into a single early-detection multimarker assay. From the available data, it appears that our results and conclusions are similar, although not identical, to those by Ciarloni et al. [25] and Cohen et al. [28].

### LB during post-surgery follow-up

Previously, Phallen et al. found a significant correlation between high ctDNA levels and poor CRC outcome [27]. Herein, we found that ctDNA is a potentially useful surrogate of tumor burden at advanced stages (it marks response to chemotherapy), and in the early post-surgery follow-up (in the absence of medical therapy). In the latter and less investigated setting, we found that regardless of absolute levels and/or presence of ctDNA at surgery, CRC patients displaying persistent ctDNA on the first post-surgery follow-up (3 months, as per standard of care) were invariably CRC-positive upon routine CT scan, whereas patients with no detectable ctDNA at surgery, at the 3 month follow-up checkpoint, or both, remained apparently disease-free for 6 or more months. Although these observations are encouraging, the low numbers do not allow drawing firm conclusions. Further studies are needed to determine whether ctDNA persistence in the immediate post-surgical follow-up may help to assign intensified adjuvant therapeutic regimens to relapse-prone, high-risk patients.

## Conclusions

In summary, our study highlights crucial differences between disease onset and advanced stages. When CRC tumors approach a critically low size threshold, the levels of individual ctDNAs may become unlinked from, or marginally dependent on, clinical pathological parameters such as size, mutation frequency and grade of the primary tumor, or nodal status. Under these conditions, uneven representation in blood of the tumor SNV load, as exemplified by our own ‘type (b)’ alterations, and/or unknown bottlenecks regulating ctDNA release may become appreciable. There is no need to postulate that these bottlenecks are specific of newly diagnosed CRC. They may operate at all disease stages but become evident only when tumor/ctDNA loads are low. Despite this difficult setting, LB at surgery is informative and accurate, and its sensitivity is likely to be readily improved through a moderate enlargement of NGS panels, so as to include sets of mutations not readily detectable in the primary. A combination of clinical NGS and dPCR, the integration of ctDNA with CEA, and the inclusion of other next generation biomarkers (miRNAs in particular) may also result in significant technical improvement and simple multimarker assays applicable to real-life oncology.

## Supplementary information

**Additional file 1: Table S1**. Patient population.

**Additional file 2: Table S2.** Clinical pathological features of the patients. Abbreviations: n.d., not determined.

**Additional file 3: Table S3.** List of dPCR assays.

**Additional file 4: Table S4.** Diagnostic performance of liquid biopsy. Assay performance were calculated for plasma samples from 39 group A patients at surgery and 10 plasma samples obtained from healthy donors. True positives (TP): CRC patients with at least one detectable (above the pre-determined assay sensitivity threshold) circulating SNV. True negatives (TN): healthy donors with no detectable circulating SNV. False negatives (FN): patients with no circulating SNV but with positive tissue. False positives (FP): SNV called in healthy donors. Specificity: n. of TN / (n. of TN + n. of FP). Sensitivity: n. of TP / (n. of TP + n. of FN). Positive predictive value: n. of TP / (n. of TP + n. of FP). Negative predictive value: n. of TN / (n. of FN + n. of TN). Accuracy: (TP + TN) / (TP + FN + FP + TN).

**Additional file 5: Fig. S1.** Mutation hotspots in tDNAs. List of mutations detected by NGS and dPCR in primary CRC tissue lesions. Only patients with detectable mutations are shown. VAF values are ranked by color intensity. Abbreviations: nt, not tested; ne, not evaluated since no amplicons were available spanning this region.

**Additional file 6: Fig. S2.** Mutation hotspots in the bloodstream. List of mutations detected by NGS and dPCR in plasma samples. Only patients with detectable mutations are shown. Mutations detected in both tissue and blood (green boxes), only in tissue (red) or only in blood (yellow) are displayed along with their corresponding VAFs. Abbreviations: nt, not tested; ne, not evaluable.

## Data Availability

Raw data supporting the findings of this study are available on the IRCCS Regina Elena National Cancer Institute website (www.ifo.it) upon request.

## Abbreviations

ACL: Ampliseq™ Colon and Lung panel
CADT: Custom Taqman® Assay Design Tool
CEA: Carcinoembryonic antigen
CRC: Colorectal cancer
ctDNA: Circulating tumor DNA
dPCR: Digital PCR
FFPE: Formalin-fixed, paraffin-embedded
LB: Liquid biopsy
mCRC: Metastatic colorectal cancer
NGS: Next generation sequencing
SNV: Single nucleotide variant
tDNA: Tissue DNA
TEC-Seq: Targeted error correction sequencing
TST: TruSight™ 15 panel
