# Supplementary material for: Cross-sectional analysis of circulating tumor DNA in primary colorectal cancer at surgery and during post-surgery follow-up by liquid biopsy
Source: J Exp Clin Cancer Res. 2020 Apr 20;39:69. doi: 10.1186/s13046-020-01569-z (PMC7168847; doi:10.1186/s13046-020-01569-z)
Supplement: Supplementary file 1 — Additional file 1: Table S1. Patient population. [file 13046_2020_1569_MOESM1_ESM.docx]

**Tab. S1. Patient population.**

| Features | Cases (%) |
| --- | --- |
| *Enrolled patients* |  |
| Early CRC | 39 (73.6) |
| Metastatic CRC | 14 (26.4) |
| *Sex* |  |
| Male | 28 (52.8) |
| Female | 25 (47.2) |
| *Site of surgery* |  |
| Colon | 25 (47.2) |
| Rectum | 13 (24.5) |
| Sigma | 8 (15.1) |
| Unknown | 7 (13.2) |
| *Histologic grade* |  |
| G1 | 3 (5.6) |
| G2 | 25 (47.2) |
| G3 | 18 (34.0) |
| Unknown | 7 (13.2) |
| *Lymph node status* |  |
| pN0 | 26 (48.1) |
| pN1 | 17 (32.1) |
| pN2 | 7 (13.2) |
| Unknown | 3 (5.6) |
| *Infiltration Tumor* |  |
| T0 | 1 (1.9) |
| T1 | 3 (5.6) |
| T2 | 7 (13.2) |
| T3 | 23 (43.4) |
| T4 | 18 (34.0) |
| Tis | 2 (3.8) |
| *Metastasis status* |  |
| M0 | 39 (73.6) |
| M1 | 14 (26.4) |
