# Supplementary material for: Cross-sectional analysis of circulating tumor DNA in primary colorectal cancer at surgery and during post-surgery follow-up by liquid biopsy
Source: J Exp Clin Cancer Res. 2020 Apr 20;39:69. doi: 10.1186/s13046-020-01569-z (PMC7168847; doi:10.1186/s13046-020-01569-z)
Supplement: Supplementary file 2 — Additional file 2: Table S2. Clinical pathological features of the patients. Abbreviations: n.d., not determined. [file 13046_2020_1569_MOESM2_ESM.docx]

| **#** | **pT** | **pN** | **M** | **Grade** | **Tumor size (cm)** | **CEA**  **(ng/ml)** | **Ca19.9**  **(U/ml)** | **cfDNA**  **(ng/ml plasma)** | **ctDNA VAF**  **(%)** |
| --- | --- | --- | --- | --- | --- | --- | --- | --- | --- |
| 1 | 3 | 2 | 1 | 1 | 3.5 | 3.2 | 1.7 | 15.7 | 0.5 |
| 2 | 1 | 0 | 0 | / | 2 | 1.2 | 10 | 16.7 | n.d. |
| 3 | 2 | 0 | 0 | 1 | 1.8 | 1.9 | 10 | 11.5 | 0.0 |
| 4 | 1 | 0 | 0 | 1 | 4.7 | 1.6 | 19.9 | 13.4 | 0.0 |
| 5 | 4 | 1 | 1 | 3 | 5 | 52.3 | 17.9 | 12.2 | n.d. |
| 6 | 4 | 2 | 1 | 3 | 2 | 2.3 | 19.3 | 30.3 | 0.0 |
| 7 | 3 | 0 | 0 | 2 | 4.5 | 4.2 | 1.2 | 47.5 | n.d. |
| 8 | 2 | 0 | 0 | 2 | 3 | 2.6 | 5.2 | 17.3 | 0.1 |
| 9 | 4 | 0 | 0 | 2 | 3 | 1.6 | 1 | 13.2 | 0.8 |
| 10 | 3 | 0 | 0 | 2 | 3 | 19.5 | 14.4 | 36.5 | n.d. |
| 11 | 3 | 0 | 0 | 3 | 5 | 0.6 | 3.5 | 12.9 | 0.4 |
| 12 | 3 | 1 | 1 | 2 | 4.5 | 0.4 | 4.4 | 24.4 | 0.4 |
| 13 | 3 | 0 | 0 | 2 | 4 | 11.8 | 29 | 38.8 | 0.0 |
| 14 | 2 | 1 | 0 | 3 | 3 | 2.9 | 26.2 | 9.7 | n.d. |
| 15 | 2 | 0 | 0 | 2 | 6 | 0.9 | 2.7 | 15.8 | n.d. |
| 16 | 2 | 0 | 0 | 2 | 3.5 | 2.9 | n.d. | 29.0 | n.d. |
| 17 | 4 | 1 | 0 | 2 | 5 | 288.4 | 0.6 | 24.3 | 1.5 |
| 18 | 3 | 1 | 0 | 2 | 4 | 1.8 | 1.5 | 26.8 | 0.0 |
| 19 | 2 | 0 | 0 | 2 | 3.5 | 2.8 | 9.7 | 54.7 | n.d. |
| 20 | 3 | 0 | 0 | 2 | 4 | 7.5 | 14.6 | 9.6 | n.d. |
| 21 | 3 | 0 | 0 | 2 | 4.5 | 1.5 | 6 | 13.8 | 0.2 |
| 22 | 3 | 0 | 0 | 2 | 4.5 | n.d. | n.d. | 23.8 | 2.2 |
| 23 | 4 | 0 | 0 | 2 | 3 | n.d. | n.d. | 7.4 | 0.4 |
| 24 | 4 | 1 | 0 | 3 | 4 | 3.5 | 22.9 | 16.9 | 0.0 |
| 25 | 3 | 0 | 0 | 3 | 5 | n.d. | n.d. | 9.7 | n.d. |
| 26 | 2 | 1 | 0 | 2 | 4 | 2.8 | 9.4 | 21.2 | 0.2 |
| 27 | 1 | 0 | 0 | 1 | 2.5 | 6.1 | 76.8 | 31.4 | 0.0 |
| 28 | 3 | 0 | 0 | 2 | 5 | 1 | 7.5 | 11.6 | 0.0 |
| 29 | 1 | 0 | 0 | / | < 1 | 0.9 | 13.0 | 8.8 | n.d. |
| 30 | 3 | 0 | 0 | 2 | 2.5 | 3 | 8.1 | 11.3 | n.d. |
| 31 | 3 | 1 | 0 | 2 | 4 | 3.4 | 26.7 | 9.4 | 0.9 |
| 32 | 4 | 2 | 0 | 3 | 7 | 10.4 | 23 | 29.0 | 7.0 |
| 35 | 4 | 0 | 0 | 3 | 5 | 9.5 | 36.1 | 12.0 | 0.0 |
| 36 | 3 | 1 | 0 | 3 | / | 12.5 | 313.8 | 11.8 | 1.6 |
| 37 | / | / | 0 | / | / | n.d. | n.d. | 28.9 | n.d. |
| 38 | 3 | 0 | 0 | 2 | 4 | 6.6 | 10.3 | 7.4 | n.d. |
| 39 | 3 | 1 | 0 | 2 | 3 | 5.7 | 13.4 | 30.6 | 0.0 |
| 41 | 3 | 1 | 0 | 3 | 4 | 0.5 | 2 | 31.6 | 3.1 |
| 42 | 3 | 1 | 0 | 3 | 5 | 7.2 | 2 | 10.3 | 0.0 |

**Tab. S2. Clinical pathological features of the patients.**
