# Supplementary figures and images for: Cross-sectional analysis of circulating tumor DNA in primary colorectal cancer at surgery and during post-surgery follow-up by liquid biopsy
Source: J Exp Clin Cancer Res. 2020 Apr 20;39:69. doi: 10.1186/s13046-020-01569-z (PMC7168847; doi:10.1186/s13046-020-01569-z)

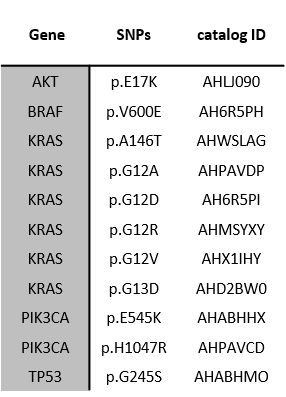

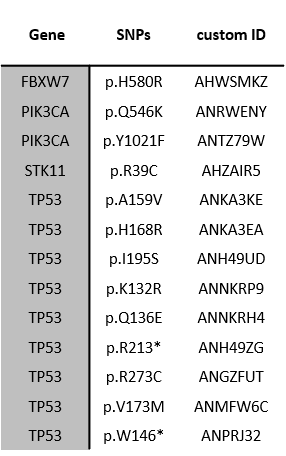


a

b

**Tab. S3. List of dPCR assays.**

Supplement: Supplementary file 3 — Additional file 3: Table S3. List of dPCR assays. [file 13046_2020_1569_MOESM3_ESM.docx]

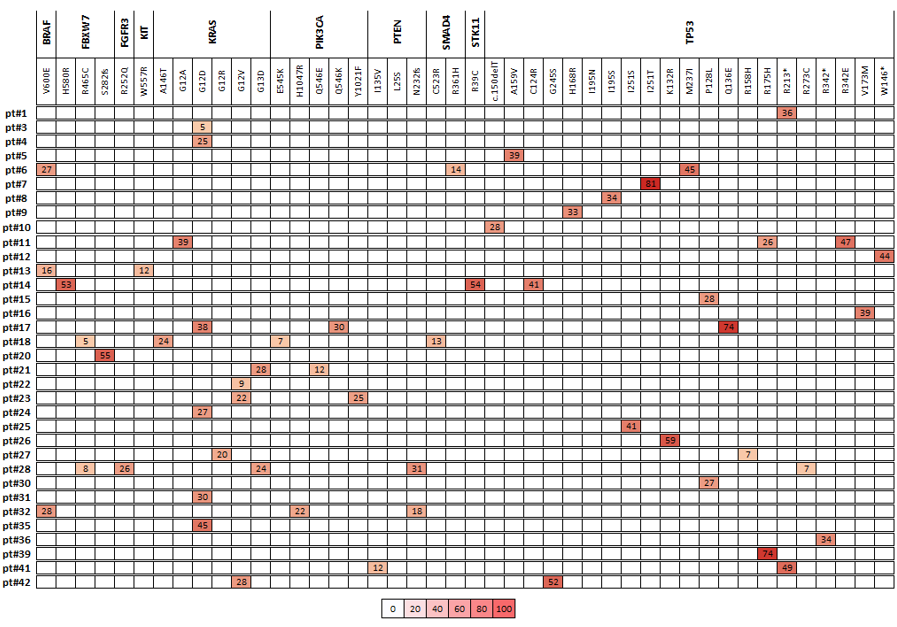

Supplement: Supplementary file 5 — Additional file 5: Fig. S1. Mutation hotspots in tDNAs. List of mutations detected by NGS and dPCR in primary CRC tissue lesions. Only patients with detectable mutations are shown. VAF values are ranked by color intensity. Abbreviations: nt, not tested; ne, not evaluated since no amplicons were available spanning this region. [file 13046_2020_1569_MOESM5_ESM.png]

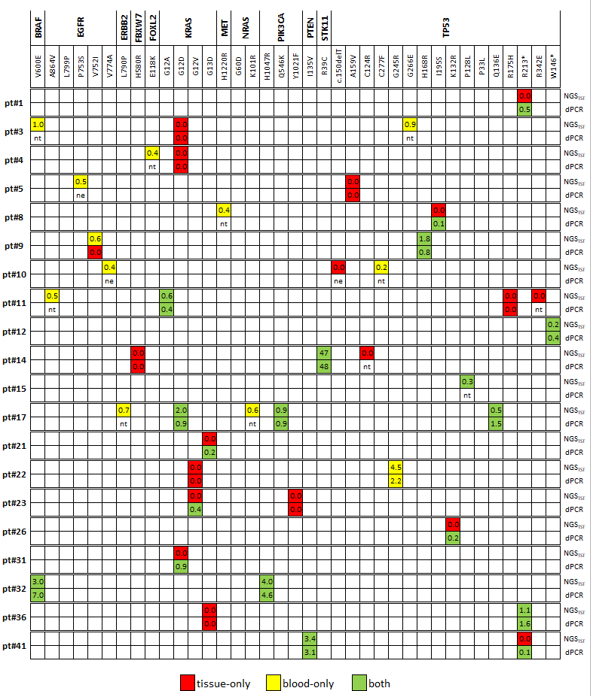

Supplement: Supplementary file 6 — Additional file 6: Fig. S2. Mutation hotspots in the bloodstream. List of mutations detected by NGS and dPCR in plasma samples. Only patients with detectable mutations are shown. Mutations detected in both tissue and blood (green boxes), only in tissue (red) or only in blood (yellow) are displayed along with their corresponding VAFs. Abbreviations: nt, not tested; ne, not evaluable. [file 13046_2020_1569_MOESM6_ESM.png]
