# Supplementary material for: Cross-sectional analysis of circulating tumor DNA in primary colorectal cancer at surgery and during post-surgery follow-up by liquid biopsy
Source: J Exp Clin Cancer Res. 2020 Apr 20;39:69. doi: 10.1186/s13046-020-01569-z (PMC7168847; doi:10.1186/s13046-020-01569-z)
Supplement: Supplementary file 4 — Additional file 4: Table S4. Diagnostic performance of liquid biopsy. Assay performance were calculated for plasma samples from 39 group A patients at surgery and 10 plasma samples obtained from healthy donors. True positives (TP): CRC patients with at least one detectable (above the pre-determined assay sensitivity threshold) circulating SNV. True negatives (TN): healthy donors with no detectable circulating SNV. False negatives (FN): patients with no circulating SNV but with positive tissue. False positives (FP): SNV called in healthy donors. Specificity: n. of TN / (n. of TN + n. of FP). Sensitivity: n. of TP / (n. of TP + n. of FN). Positive predictive value: n. of TP / (n. of TP + n. of FP). Negative predictive value: n. of TN / (n. of FN + n. of TN). Accuracy: (TP + TN) / (TP + FN + FP + TN). [file 13046_2020_1569_MOESM4_ESM.docx]

| True positives (n.) | 20 |
| --- | --- |
| False positive (n.) | 0 |
| True negative (n.) | 10 |
| False negative (n.) | 19 |
| Positive Predictive Value (%) | 100.0% |
| Negative Predictive Value (%)  (95% CI) | 34.5 %  (27.6% to 42.1%) |
| Sensitivity (%)  (95% CI) | 51.3%  (34.8% to 67.6%) |
| Specificity (%)  (95% CI) | 100.0 %  (69.2% to 100.0%) |
| Accuracy (%)  (95% CI) | 61.2%  (46.2% to 74.8%) |

**Tab. S4. Diagnostic performance of liquid biopsy.**
